# Positive selection drives the evolution of endocrine regulatory bone morphogenetic protein system in mammals

**DOI:** 10.18632/oncotarget.24240

**Published:** 2018-01-13

**Authors:** Hafiz Ishfaq Ahmad, Muhammad Jamil Ahmad, Muhammad Muzammal Adeel, Akhtar Rasool Asif, Xiaoyong Du

**Affiliations:** ^1^ Key Laboratory of Agricultural Animal Genetics, Breeding and Reproduction of the Ministry of Education, College of Animal Science and Technology, Huazhong Agricultural University, Wuhan 430070, P.R. China; ^2^ University of Veterinary and Animal Sciences, Lahore, Sub Campus Jhang, Pakistan; ^3^ Hubei Key Laboratory of Agricultural Bioinformatics, College of Informatics, Huazhong Agricultural University, Wuhan 430070, P.R. China

**Keywords:** selection, BMPs, nucleotide substitutions, maximum likelihood, evolution

## Abstract

The rapid evolution of reproductive proteins might be driven by positive Darwinian selection. The bone morphogenetic protein family is the largest within the transforming growth factor (TGF) superfamily. A little have been known about the molecular evolution of bone morphogenetic proteins exhibiting potential role in mammalian reproduction. In this study we investigated mammalian bone morphogenetic proteins using maximum likelihood approaches of codon substitutions to identify positive Darwinian selection in various species. The proportion of positively selected sites was tested by different likelihood models for individual codon, and M8 were found to be the best model. The percentage of positively elected sites under M8 are 2.20% with ω = 1.089 for BMP2, 1.6% with ω = 1.61 for BMP 4 0.53% for BMP15 with ω = 1.56 and 0.78% for GDF9 with ω = 1.93. The percentage of estimated selection sites under M8 is strong statistical confirmation that divergence of bone morphogenetic proteins is driven by Darwinian selection. For the proteins, model M8 was found significant for all proteins with ω > 1. To further test positive selection on particular amino acids, the evolutionary conservation of amino acid were measured based on phylogenetic linkage among sequences. For exploring the impact of these somatic substitution mutations in the selection region on human cancer, we identified one pathogenic mutation in human BMP4 and one in BMP15, possibly causing prostate cancer and six neutral mutations in BMPs. The comprehensive map of selection results allows the researchers to perform systematic approaches to detect the evolutionary footprints of selection on specific gene in specific species.

## INTRODUCTION

The bone morphogenetic protein family is the largest within the transforming growth factor (TGF) superfamily and the distinguished structural feature of TGF superfamily is the presence of seven conserved cysteine, which are involved in folding of molecule into distinct three dimensional structure called cysteine knot [1]. Recent studies revealed that BMP is an important component of regulatory system in endocrine tissues and various BMP functions have been observed in ovaries, pituitary and adrenal glands [2] and also a role in bone formation or differentiation [3]. Therefore it is likely that recruitment, selection and atresia of developing follicles, ovulation and lutenization or luteolysis are escorted by spatial and temporal changes in BMP genes expression pattern [2]. BMP4 expressed in theca cells enhance follicle stimulating hormone (FSH) induced estradiol production and reduce production of progesterone [4]. BMP2 and 4 enhance FSH induced estradiol production by stimulating FSH induced mitogen activated protein kinase (p38-MAPK) phosphorylation [5]. These findings have been extended to over expression studies in mouse [6], Xenopus [7], and chick [8], which indicates that BMP3 negatively regulates the BMPs and activin pathways, while the defined mechanism of inhibition is still unclear. BMP15 is solely expressed in oocytes [9, 10] and has been found to induce granulose cell mitosis and reduce FSH action by inhibiting follicle stimulating hormone receptor expression [11]. FSH induced expression of 3β-hydroxysteroid dehydrogenase, luteinizing hormone receptor subunits, steroidogenic acute regulatory protein, and steroid side chain cleavage enzyme (p450scc) all are suppressed by BMP15 [11]. The findings revealed that FSH induced progesterone synthesis is inhibited by BMP15, like BMP4, BMP6, BMP7 and GDF9, BMP15 is also a part of luteinization inhibitors group [12]. BMP15 also stimulates the expression of kit ligand mRNA in granulose cells [13]. BMP ligands and receptors are also expressed in adult pituitary glands, specifically BMP6, 7 and 15 mRNAs are expressed in mice pituitary [14], GDF9 mRNA in human pituitary, BMP15 and GDF9 mRNAs in brush tail opossum and BMP15 in sheep pituitaries [11]. Codon based likelihood models have been extremely used in recent development and have proven remarkably useful in selective pressure studies in various systems [15, 16]. An unequivocal evidence of positive selection in molecular evolution is remarkably higher in non-synonymous than synonymous substitution rate and the ratio dN/dS indicated here by ω, quantifies the magnitude and direction of selection pressure on protein, with ω = 1, ω = > 1, and ω = < 1 indicate neutral evolution, positive selection and purifying selection respectively [17]. These conditions have been used to study evolution of male reproductive protein in vertebrate and invertebrate species [18]. To expound the selection pressure underlying the rapid evolution of bone morphogenetic proteins, herein we perform an analysis revealing that positive Darwinian selection drives the evolution of bone morphogenetic proteins in several mammals. We revealed that positive selection has driven on bone morphogenetic proteins as evidenced by population genetic signals such as greater number of non-synonymous substitution rates, long range of haplotype homozygosity and lower genetic diversity. Our results support the hypothesis that there was a rapid evolutionary pressure on mammalian bone morphogenetic proteins genes during evolution.

## RESULTS

The ω ratios for all bone morphogenetic proteins across the sites are < 1 (Table 1). However, these proteins might have conserved amino acid and are showing purifying selection with ω < 1. The conserved amino acids might mask the signals of positive selection and adaptable amino acids showed positive selection, that were exposed or hidden residues according to the neural network algorithm for BMP2, BMP4, BMP15 and GDF9 (Supplementary Figures 1–4 respectively). The dN/dS ratio was an average of all positions, and so average dN/dS could not identify the positive selection precisely [24, 25]. Hence, the positive selection was tested particular amino acids using ML tests that measure selective pressures among sites stated by ω values [19, 20].

**Table 1:** Model parameter estimates, dN/dS ratios, log likelihood values and test statistics for PAML site models of positive selection in mammalian bone morphogenetic proteins

Gene	n	Lc	S	dN/dS	Model	Parameter estimates	2ΔlM2 vs. M1	2ΔlM8 vs. M7	Positively selected sites
BMP2	39	398	3.5	0.08	M1	P1 = 0.92674 P2 = 0.07326	0	8.14^*^	37, 38, 120, 126, 162, 183, 190, 239
						ω1 = 0.04427 ω2 = 1.00000			
					M2	P1 = 0.92674 P2 = 0.05049 P3 = 0.02277			
						ω1 = 0.04427 ω2 = 1.00000 ω3 = 1.00000			
					M7	p = 0.16499 q = 1.54264			
					M8	P0 = 0.97794 p = 0.21124 q = 2.75784			
						**P1 = 0.02206 ω1 = 1.08959**			
GDF9	33	457	8.1	0.31	M1	P1 = 0.59348, P2 = 0.40652	0.48	2.59	30, 186, **245**, 254, **292**, 302, 304
						ω1 = 0.12471, ω2 = 1.00000			
					M2	P1 = 0. 59353, P2 = 0. 33608, P3 = 0. 07040			
						ω1 = 0. 12473, ω2 = 1.00000, ω3 = 1.00000			
					M7	p = 0. 48463 q = 0. 91798			
					M8	P0 = 0. 99219 p = 0. 49979 q = 0. 97580			
						**P1 = 0.00781, ω1 = 1.93407**			
BMP4	29	570	4.3	0.09	M1	P1 = 0.93285, P2 = 0.06715	3.41	26.91^**^	**255, 256**, 259, 333, **349**, 351, 375, 425
						ω = 0.05872, ω1 = 1.00000			
					M2	P1 = 0.93307, P2 = 0.06381, P3 = 0.00312			
						**ω1 = 0.05939, ω2 = 1.00000, ω3 = 2.87878**			
					M7	p = 0.23270 q = 1.77764			
					M8	P0 = 0.98531 p = 0.31720 q = 3.28217			
						**P1 = 0.01469 ω1 = 1.61688**			
BMP15	86	434	13.5	0.41	M1	P1 = 0.54292 P2 = 0.45708	8.4^*^	17.69^**^	31, 37, 89, **113,** 154, **169**, 177, 178, 229, 285
						ω1 = 0.17179 ω1 = 1.00000			335
					M2	P1 = 0.53569 P2 = 0.44200 P3 = 0.02231			
						**ω1 = 0.17280 ω2 = 1.00000 ω3 = 2.12476**			
					M7	p = 0.60414 q = 0.76298			
					M8	P0 = 0.94697 p = 0.66983 q = 0.97138			
						**P1 = 0.05303 ω1 = 1.56019**			
						P1 = 0.21259 ω1 = 1.00000			

The data have *n* sequences, each of *L*c codons after alignment gaps are removed. *S* is the tree length, measured as the number of nucleotide substitutions per codon. The proportion of sites under positive selection (*p*1), or under selective constraint (*p*0), and parameters *p* and *q* for the beta distribution. Parameters indicating positive selection are in bold. p: significant at 5% level; pp: significant at 1% level. Sites potentially under positive selection identified under model M8 are listed according to the human sequence numbering. Positively selected sites with posterior probability 0.9 are underlined, 0.8–0.9 in bold, and 0.5– 0.7 in plain text. The test statistic *2Δl* is compared to a χ^2^ distribution with 2 degrees of freedom, critical values 5.99, 9.21, and 13.82 at 5%, 1%, and 0.1% significance, respectively. ^**^: significant at 1% level; ^*^: significant at 5% level.

### Bone morphogenetic proteins

Positive selection was found in BMP2, BMP4, BMP15 and GDF9. We performed log likelihood test for all BMP proteins and the ω was estimated for all sites. We compared various likelihood tests (M1 vs. M2, and M7 vs. M8 respectively) to determine positive selection. Parameter estimates under M1 and M2 were compared and there was positive selection in M2 for two of four proteins. The proportions of positive selection sites were 0.31% with ω = 2.87 for BMP4 and 2.23% with ω = 2.12 for BMP15 (Table 1). M8 was significant for all bone morphogenetic proteins. The percentage of positively selected sites under M8 are 2.20% for BMP2 with ω = 1.089, 0.78% for GDF9 with ω = 1.93, 1.6% for BMP4 with ω = 1.61 and 0.53% for BMP15 with ω = 1.56.

### Positive selection on amino acids

**Figure 1:**
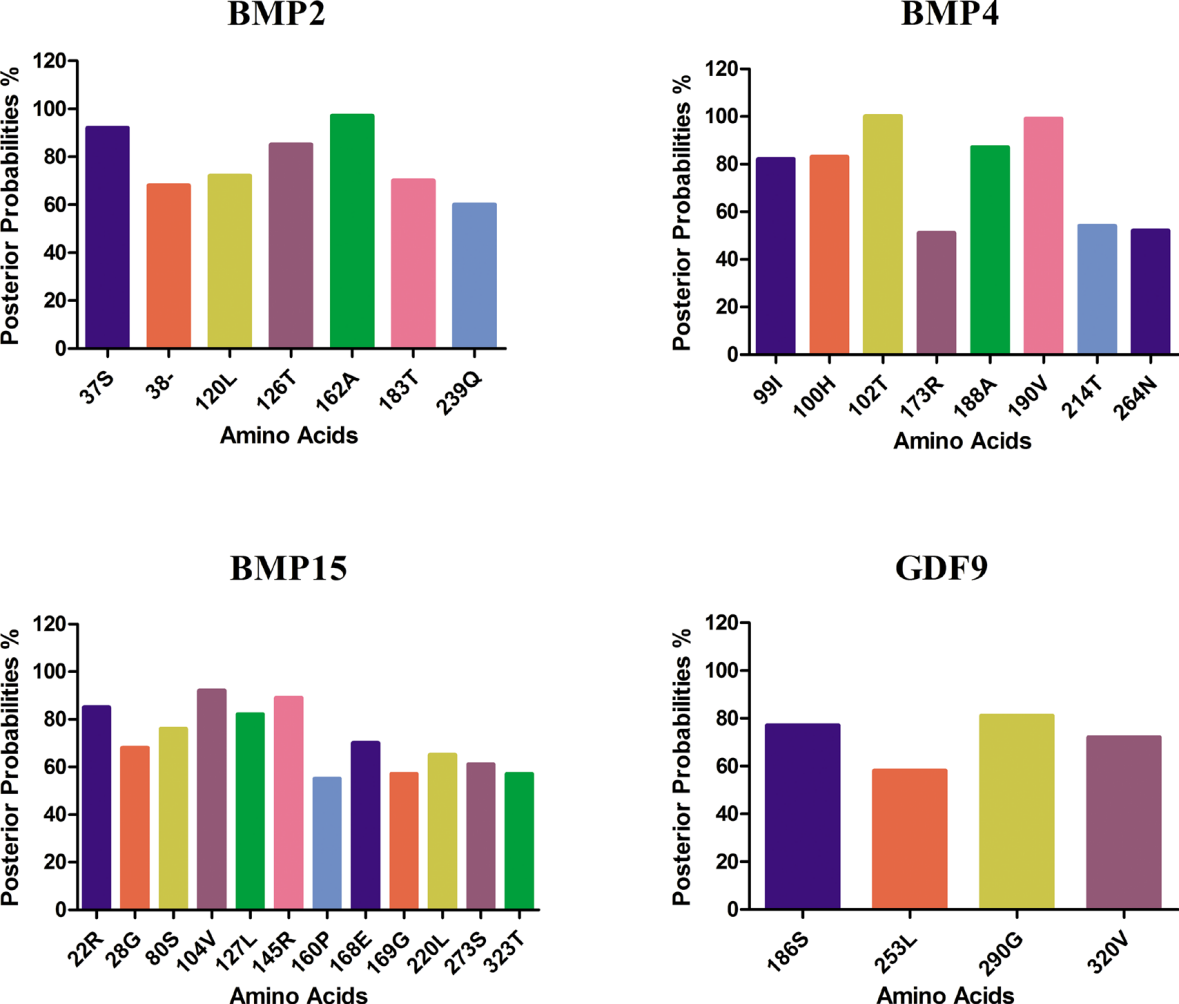
Amino acid residues identified likely to be under positive selection by bayes empirical bayes The amino acid sites of ω > 1 under M8 model. The posterior probability of each site was calculated by BEB. Sites show positive selection at different posterior probabilities.

**Figure 2:**
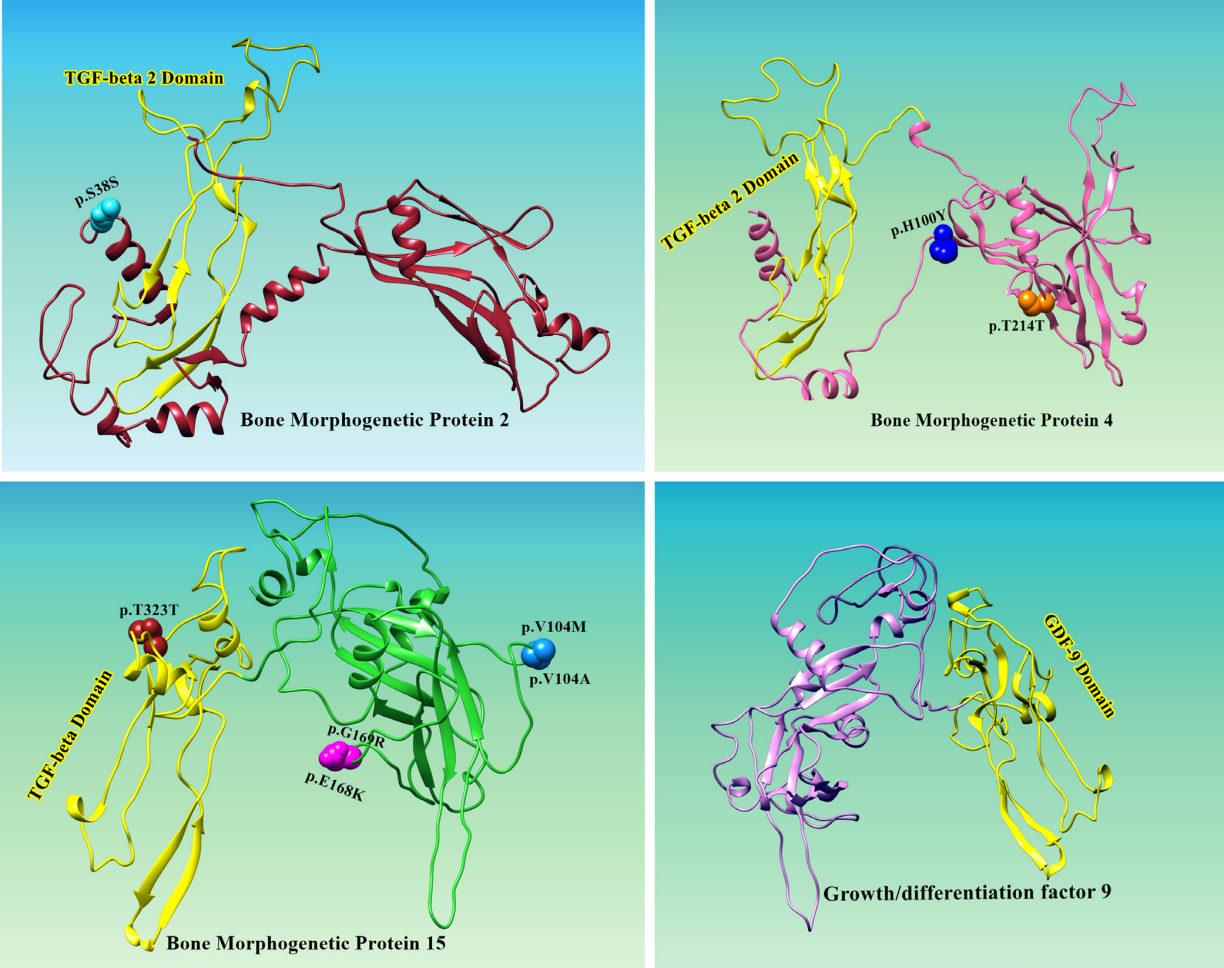
Location of positively selected amino acid sites identified BMP2, BMP4, BMP15 and GDF9 genes Three dimensional structure prediction of BMPs and GDF9 was carried out by using Ab-initio modeling approach. Primary sequences of human BMP2 (ACV32596.1), BMP4 (AAH20546.1), BMP15 (AAI17265.1) and GDF9 (AAH96229.1) were subjected to I-TESSAR to predict suitable structures. Structure validation of all predicted models was done by MolProbity server. To test the steric hindrance of amino acid residues Ramachandran values were calculted by using Ramachandran Plot2.0 tool. UCSF Chimera was applied for visualization and geometry optimization of predicted proteins. All the residues identified as under selection fall in the domain containing the ligand binding site. The sites which fall in the region identified as the ligand binding site and another cluster in a region immediately following the signal sequence.

To identify amino acid positions under selection pressure, we used the Bayes approaches to approximate the posterior probabilities for individual codon position. The codon with higher probabilities is likely to be under positive selection with ω > 1 [25]. Using Bayes Empirical Bayes (BEB) analysis for BMP2 had a total of 391 amino acids sites, and seven sites were detected under positive selection (Table 2; Figure 1). Only one of the seven sites has posterior probability > 95% and the position of site is shown in protein structure (Figure 2). GDF9 has four hundred and fifty three amino acids, and only seven were found under positive selection and BMP4 had 401 amino acids, and eight were found under positive selection (Figure 2). Two of these eight sites are positively selected at posterior probability > 99% and 95% respectively (Table 2; Figure 1). As well BMP15 has three hundred and ninety one amino acid of seventeen positive selection sites but no codon site could be recognized at 99% or 95% posterior probabilities (Table 2; Figure 1).

**Table 2:** Positively selected sites under different PAML site models using bayes empirical bayes analysis

Gene	Model	Codon	Amino Acid	Posterior Probability	Post mean ± SE for ω
BMP-2	M8: selection,	38	S	0.695	1.187 ± 0.532
	beta+ ω	41	P	0.632	1.114 ± 0.554
		43	S	0.713	1.230 ± 0.472
		118	L	0.597	1.079 ± 0.555
		164	N	0.611	1.087 ± 0.569
		236	K	0.607	1.115 ± 0.518
GDF-9	M8: selection,	186	S	0.585	1.225 ± 0.335
	beta+ ω	253	L	0.696	1.300 ± 0.309
		290	G	0.832	1.395 ± 0.238
		302	V	0.938^*^	1.463 ± 0.148
BMP-4	M8: selection,	99	I	0.823	1.368 ± 0.311
	beta+ ω	100	H	0.827	1.370 ± 0.317
		102	T	0.998^**^	1.512 ± 0.123
		173	R	0.506	1.075 ± 0.449
		188	A	0.867	1.401 ± 0.309
		190	V	0.986^*^	1.503 ± 0.143
		214	T	0.536	1.071 ± 0.488
		264	N	0.515	1.073 ± 0.461
BMP-15	M8: selection,	22	R	0.590	1.239 ± 0.368
	beta+ ω	28	G	0.753	1.361 ± 0.332
		80	S	0.544	1.198 ± 0.392
		104	V	0.846	1.426 ± 0.285
		127	L	0.514	1.393 ± 0.236
		145	R	0.764	1.369 ± 0.322
		160	P	0.615	1.255 ± 0.376
		168	E	0.703	1.315 ± 0.291
		169	G	0.759	1.365 ± 0.329
		220	L	0.556	1.212 ± 0.373
		273	S	0.547	1.198 ± 0.397
		323	T	0.717	1.334 ± 0.339

Bayes Empirical likelihood ratio test statistic for model M8: selection, beta+ ω, indicate posterior probability P > 95% (^*^) and P > 99% (^**^). For codon position, the amino acid number is given followed by an abbreviation.

One neutral single nucleotide polymorphism p.S38S (score 0.15) was found in BMP2 and one pathogenic mutations causing prostate cancer, p.T214T was identified in BMP4 (Table 3). p.S38S is found in the functionally quiet pro-domain of BMP2 and is predicted by FATHMM and to be benign/tolerated. p.T214T is expected to be possibly detrimental or to disturb the protein structure. However, a basic amino acid residue at position 38 (S) is essential for recognition or cleavage of site. Serine is a polar amino acids and p.S38S is therefore suspected to have influence on post-translational cleavage and biosynthesis of protein. This mutation is anticipated to be benign or tolerated by FATHMM (Table 3). Only one mutation, p.T214T was identified genomic region of BMP4 encoding functional domain of TGF-β.

**Table 3:** Prediction of pathogenic point amino acid substitutions mutation was estimated from the usage of functional analysis through hidden Markov model (FATHMM)

Gene	Codon	Snp Id	Tissue Distribution	FATHMM prediction: (Functional Analysis through Hidden Markov Models)
BMP-2	38	COSM1029277	Endometrium(1)/Prostate(1) p.S38S	Neutral (score 0.15)
	41			
	43			
	118			
	164			
	236			
GDF-9	186			
	253			
	290			
	302			
BMP-4	99			
	100	XXX	Lung p.H100Y	Pathogenic (score 0.98)
	102			
	173			
	188			
	190			
	214	XXX	Hematopoietic and lymphoid tissue(1)/Prostate(1) p.T214T	Pathogenic (score 0.84)
	264			
BMP-15	22			
	28			
	80			
	104	COSM4649428	Large intestine(1) p.V104M	Neutral (score 0.03)
	104	COSM6187212	Lung(1) p.V104A	Neutral (score 0.03)
	127			
	145			
	160			
	168	COSM385794	Lung(1) p.E168K	Neutral (score 0.10)
	169	COSM3562207	Skin(1) p.G169R	Neutral (score 0.01)
	220			
	273			
	323	COSM309487	Lung(2) p.T323T	Neutral (score 0.01)

The codon sites that have undergone alteration of gene can direct to more chances of false positive results during analysis when using ML approach to identify positive selection, predominantly in small data set which only a few sequences, though false positive rate is moderately increased [21]. To reduce the gene conversion influence on results of the study, each set of sequence was analyzed individually and the most similar sequences, which are the results of gene conversion were excluded from the analysis. Furthermore, a BEB analysis was used instead of NEB to recognize positively selected codon because NEB is less conservative and can be more exposed to false positive results [22, 24]. While BEB is more conservative and yields a less chances of false positive results with codon sites that are influences by gene conversion [21].

## DISCUSSION

Recently, male derived molecules have been shown to be extraordinarily involved in reproduction between closely related species, which include proteins involved in signaling between males and females fertilization. Preceding studies using ovarian theca interna cell cultures revealed that various BMPs (BMP2, 4, and 6) potentially suppress LH induced androgen secretion [23]. BMPs along with their receptors and extracellular binding proteins (e. g., chordin, noggin and gremlin) are extensively expressed in granulosa, theca cells and ovarian stroma [24, 25]. It is likely that BMPs exert autocrine as well as paracrine action to regulate steroidogenesis and other ovarian functions [26]. Previous studies directed us to hypothesize that evolution of BMPs has emerged implicating a variety ovarian factors having modulatory role in reproduction process. As recently in evolution, the mature domains of BMP ligands BMP2, 4, 6, and 7 shares 40% amino acid identity in phylogenetic clade [27]. For most of BMP sites under positive selection, a correlation was found among the sites and interaction with other molecules. To validate the correlation between BMPs and their functions, we detected positive selection in BMP2, 4, 15 and GDF9 based on ω (dN/dS) ratio, is useful for estimation of selection pressure of genes [22, 23]. The ω > 1 indicates positive selection [28]. In our study positive selection was found with ω > 1 in BMP2, 4, 15 and GDF9 (Table 1). This shows that non-synonymous (dN) positions evolved faster than those of synonymous positions and the Darwinian selection effect purifying or balancing selection preferred new variations and higher allelic polymorphism [29] that might insert new variations in protein structure confirmation, thus disturbing the signaling pathways [30]. The amino acid replacements among species might be a result of distinct deviation from their shared ancestries which agrees with previous studies [31] that as orthologs vary from their most recent common ancestors, their different evolutionary routes direct to deviation in the discerning restraints on homologous sites [32].

In this study we predicted the linkage between germ line mutation in BMPs and risk of prostate cancer. We identified one pathogenic mutation, p.T214T in BMP4, causing prostate cancer and one neutral mutation p.S38S in BMP2. p.T214T localizes at N-terminal of TGF-β1 pro-domain pruning the protein prior to active domain. BMP4 play a role in the osteogenesis of the PCa-118b xenograft. The confirmation for this declaration is provided by the fact that deactivating mutations in SMAD 4 and BMPR1A [33], that are members of the TGF-β superfamily and cause prostate cancer. Although the bone forming phenotype in prostate cancer, bioinformatics analysis of the mutations identified as pathogenic in BMP4 and exposed that both osteoblastic and osteolytic lesions are present in the same loci. These facts provide strong indication that haplo-insufficiency of BMP4 will elevates PCa risk. About 70% of missense mutations have confirmed the presence 1% of population frequencies [34]. While we identified one neutral and one pathogenic mutation in BMP2 and BMP4 correspondingly mapped on pro-domain of the expressed protein. The cumulative risk of PCa is a result of variants causing rare disorder. The usage of various tools for next generation sequencing approaches, to screen PCa causative variants will need expression of variants through bioinformatics techniques.

Three sites were found under positive selection using the maximum likelihood model. The conventional models M1 vs. M2 comparison did not show significance for BMP2 and GDF9 but it was statistically significant for BMP4 and BMP15 identified 0.31% and 2.23% positive selection with ω values ω3 = 2.87 and 2.12 respectively (Table1). The results achieved under different sets of models (M1 vs. M2 and M7 vs. M8) vary in some aspects. While the M7 vs. M8 comparison show significant difference from former models, allowing positive selection for BMP2, 4, 15 and GDF9. For BMP4 and BMP15, both M1 vs. M2 and M7 vs. M8 were significant, the comparison detected same eight sites for BMP4 and seventeen codon sites for BMP15 had been identified under M1 vs. M2 comparison. Additionally, the positive selection signals were tested in birds and reptiles [35]. The observed positive selection in birds was a distinctive signal and not a pervasive trend, since only ~22% of genes exhibited sign of positive selection in reptiles. Likewise, the presence of positive selection shown different targets bone-associated genes in different clades. Of the 89 genes, ~18% were detected under positive selection in both mammals and birds, only 12.4% were identified in only mammals and 34.8% sites in genes encoding proteins were found under positively selection in birds and involved in bone resorption [36]. The variations in the results found using various models revealed that M1 vs. M2 comparison is more conservative test which may unable to identify positively selected sites detected by less conservative models M7 vs. M8 comparisons [19, 20]. It is remarkable to find that for BMP2 and BMP4, the amino acid sites that were detected having experience of positive selection are located mature extracellular domains of BMPs receptors which are involved in oocyte maturation and early embryonic development [37, 38]. BMP receptors composed of extra cellular domains, membrane bound domains and intracellular domains with active serine and threonine regions [39]. BMPs initiate signal transduction cascade by forming heterodimer complex through binding cell surface receptors and this complex consists of serine and threonine kinase receptors [40]. Three of four types of receptors present in TGF-b family interact with BMPs for example; BMP2 and BMP4 bind type I receptors and recruit type II receptors, while BMP6 and BMP7 interact with type II and recruit type I receptors [37, 38]. Moreover the distinct expression patterns of BMP4 and BMP6 mRNA in different systems propose that BMPs are involved in events that control embryonic development pattern [41]. Although the signaling pathways for BMP ligands have been studied, there are alternative pathways for BMPs that mediate biological activity in various cell types [42], particularly the MAPK signaling molecules family, including p38, ERK1/2 and N-terminal kinases have been shown to exhibit intracellular transduction of BMP signal pathways which regulate granulosa cells function [43].

Selection pressures occurring indifferent lineages may result in parallel or convergent alterations at amino acid site refer to amino acids changes from different ancestral to the same descendant amino acid along independent evolutionary lineages [44, 45]. Among the positively detected sites we observed that all sites fall in extracellular ligands binding domains for a prodomain folding and C-terminal mature peptide except for five sites of which one for BMP2 (37S) and four for BMP15 (40V, 49I, 75Q and 90R) were at N-terminus (Figure 2; BMP2, BMP15) and C-terminal proteins are cleaved proteolytically upon dimerization at an Arginine sequence by serine endoprotease from prodomain [46]. Previous studies revealed that BMPs are consist of 50 to 100 amino acids with seven cysteine, of which six form cysteine knot and the seventh cysteine is used for dimerization, thus developing the biologically active signaling molecules [47]. All the BMPs; BMP2, 4, 6 and 7 have seventh cysteine and form homodimer or heterodimer except BMP3 and BMP15 lack seventh cysteine but are biologically active as monomers [48, 49]. Among molecular level positive selection reported in previous studies favors more abundant non-synonymous substitutions and here we detected positive selection having all non-synonymous substitutions that occurred as results of duplication of genes during evolution in mammals [50, 51].

## MATERIALS AND METHODS

### Sequence analysis and dataset preparation

The nucleotide and amino acid sequences of BMP genes retrieved from GenBank (www.ncbi.nlm.nih.gov/genbank) (Supplementary Table 1) and accomplished sequences of proteins were aligned using the MUSCLE [52], implemented in MEGA6.0 program using the amino acid sequences and back translated to nucleotide for selection analysis [53].

### Positive selection analysis

In order to identify codons under positive selection, only BMPs and GDF9 that were represented in at least 20 species were assessed, as conferred by poon and collaborators [54]. Hence, BMP6 and BMP7 were excluded from analyses as the multiple sequence alignment generated were not reliable and prone to affect the recognition of selection, leading false positive results [55]. Phylogenetic analysis was performed on accepted mammalian phylogeny [56] by generating un-rooted tree of aligned species. Branch lengths were calculated using tree topology using the codon model in PAML package. The different ω ratios (dN/dS) were compared to identify selection pressure in particular codons using maximum-likelihood methods implemented in the MEGA6 [54] and PAML version 4 [57].

We compared different likelihood ratio tests. The M7 (null model) assumes β distribution with ω in limited (0 and 1) interval. The M8 is an alternative model that includes two parameters (ω and beta), so ω value achieved from the data were greater than one. Additionally, to find out amino acid exposed to selection were inferred using Bayes theorem by estimating posterior probabilities for each site [57, 58]. Three dimensional structure prediction of BMPs and GDF9 was carried out by using Ab-initio modeling approach [59]. Primary sequences of BMP2 (ACV32596.1), BMP4 (AAH20546.1), BMP15 (AAI17265.1) and GDF9 (AAH96229.1) were subjected to I-TESSAR [60] to predict suitable structures. Structure validation of all predicted models was done by MolProbity server [61]. To test the steric hindrance of amino acid residues Ramachandran values were calculted by using Ramachandran Plot2.0 tool [62]. UCSF Chimera [63] was applied for visualization and geometry optimization of predicted proteins. The ConSurf server was used to predict the level of evolutionary conservation amino acid sites in protein based on phylogenetic linkage among sequences [64]. For a more traditional approach, and as used previously [65], positive selection sites detected in more than one maximum likelihood approach were considered. We found that the statistical approaches used in this study are able to determine positive selection, but cannot deliver information about positive selection mechanism. Therefore, to printout the location of positively selected amino acid residues might be helpful for additional laboratory examination.

For these coding sites subjected to positive selection, we used the COSMIC (Catalogue of Somatic Mutations in Cancer) v82 (released 03-AUG-17) database for exploring the impact of these somatic substitution mutations in human cancer [66]. The COSMIC database includes hundreds of thousands of human cancer-associated somatic mutations that are classified by tumor type and disease. The prediction of pathogenic point amino acid substitutions mutation was estimated from the usage of Functional Analysis through Hidden Markov Model (FATHMM) [67].

## CONCLUSIONS

Our study reveals the advantages in combining various approaches to explore selection pressure and molecular evolution in biological systems, predominantly in genes intensely knotted to ecology, and highlights the significance of studies integrating natural sequence variation in organisms from various environments. Selection studies of bone morphogenetic proteins could expedite the improvement of distinctive approaches. These methodologies could possibly play a vital role for selection of higher breeding values and that their genetic enrichment to produce next generation.

## SUPPLEMENTARY MATERIALS FIGURES AND TABLES




