# Supplementary material for: Positive selection drives the evolution of endocrine regulatory bone morphogenetic protein system in mammals
Source: Oncotarget. 2018 Jan 13;9(26):18435–45. doi: 10.18632/oncotarget.24240 (PMC5915083; doi:10.18632/oncotarget.24240)
Supplement: Supplementary file 1 [file oncotarget-09-18435-s001.pdf]

Positive selection drives the evolution of endocrine regulatory bone morphogenetic protein system in mammals

SUPPLEMENTARY MATERIALS

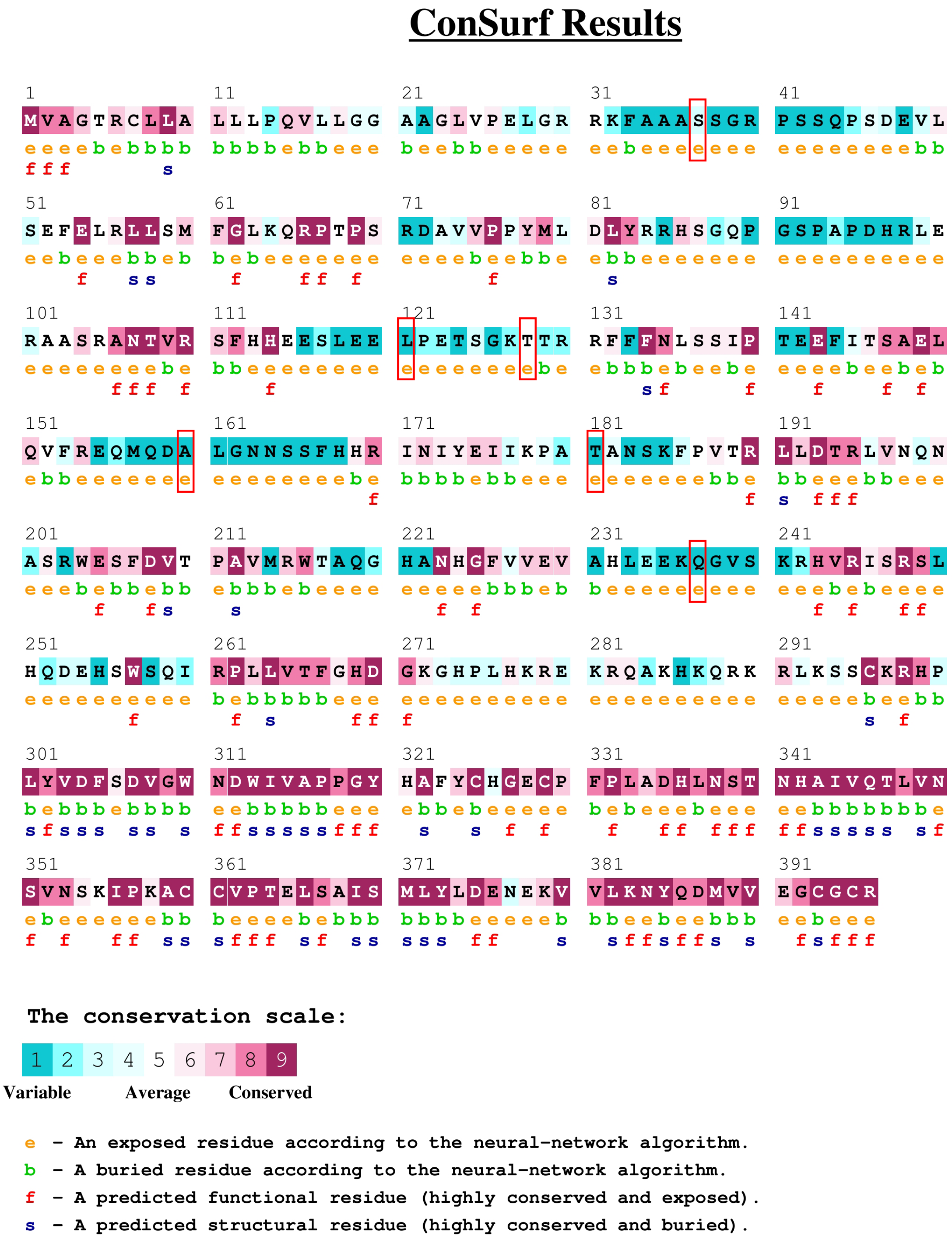

Supplementary Figure 1: ConSurf output of BMP2, using the UniRef90 protein database. Colors of the ConSurf output indicate the level of sequence conservation. Purple indicates conservation and blue indicates variability. Residues are predicted to be exposed (**e**), buried (**b**), highly conserved and exposed (**f**), or highly conserved and buried, (**-**).

ConSurf Results

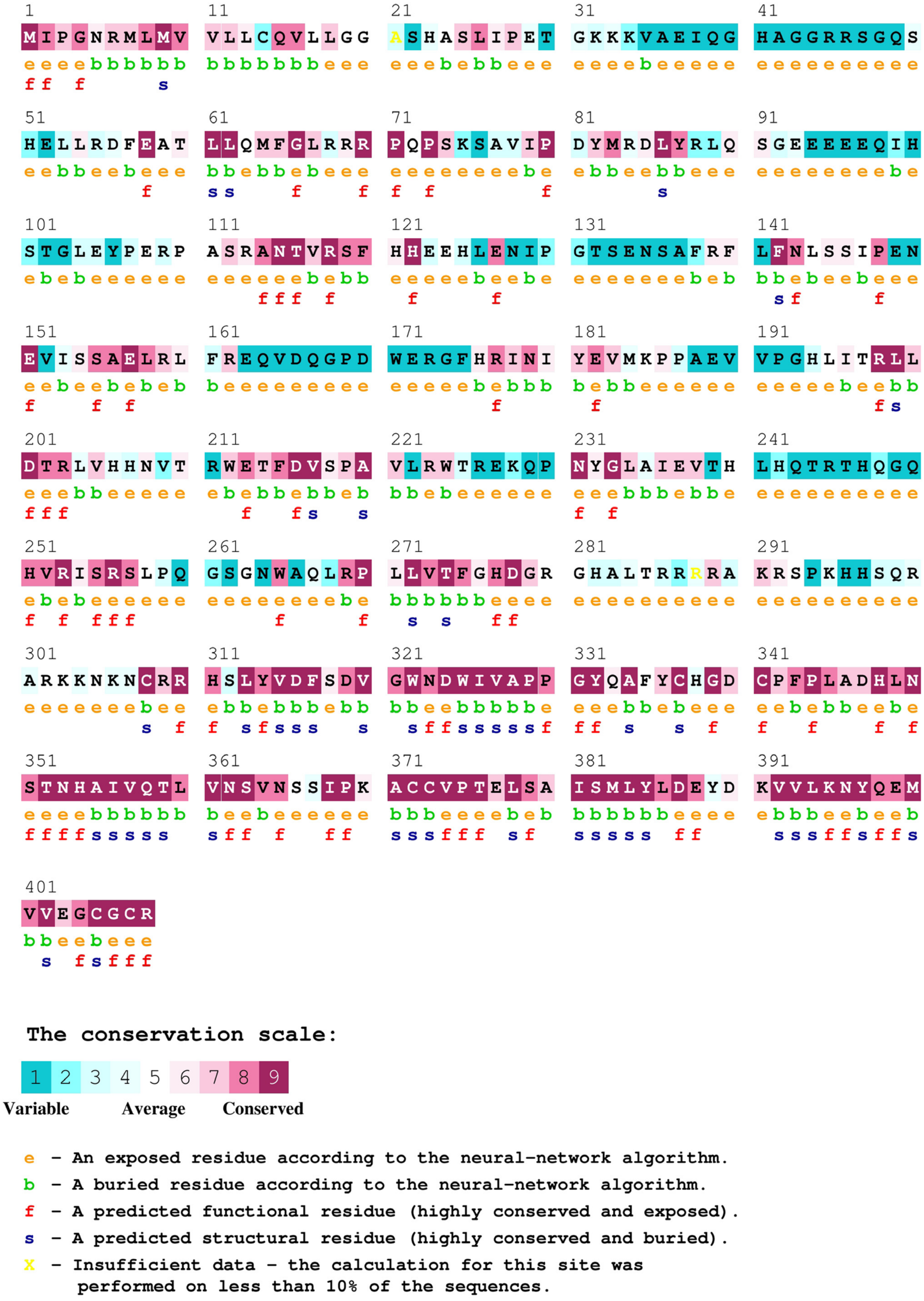

Supplementary Figure 2: ConSurf output of BMP4, using the UniRef90 protein database. Colors of the ConSurf output indicate the level of sequence conservation. Purple indicates conservation and blue indicates variability. Residues are predicted to be exposed (**e**), buried (**b**), highly conserved and exposed (**f**), or highly conserved and buried, (**s**).

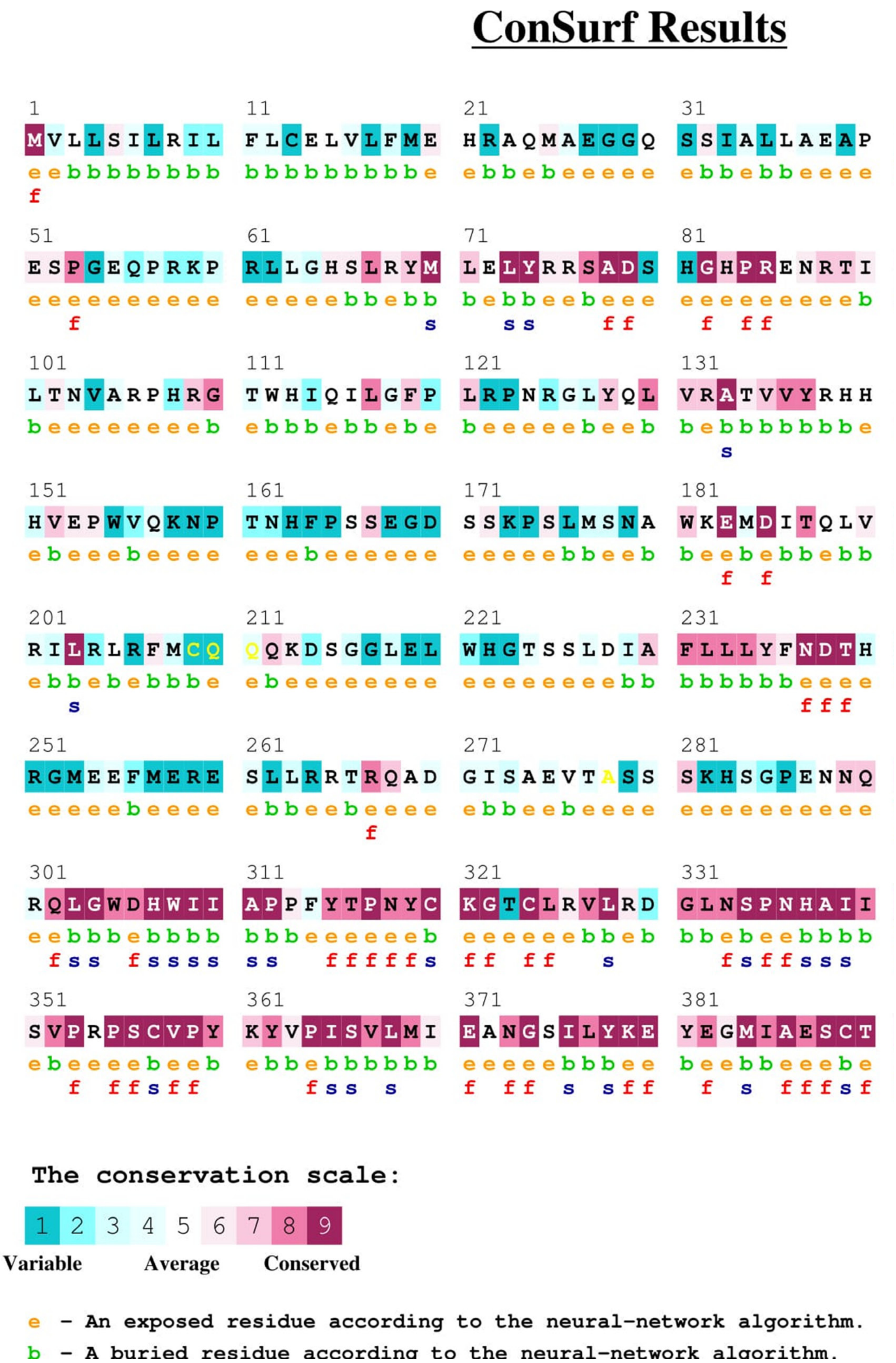

Supplementary Figure 3: ConSurf output of BMP15, using the UniRef90 protein database. Colors of the ConSurf output indicate the level of sequence conservation. Purple indicates conservation and blue indicates variability. Residues are predicted to be exposed (**e**), buried (**b**), highly conserved and exposed (**f**), or highly conserved and buried, (**s**).

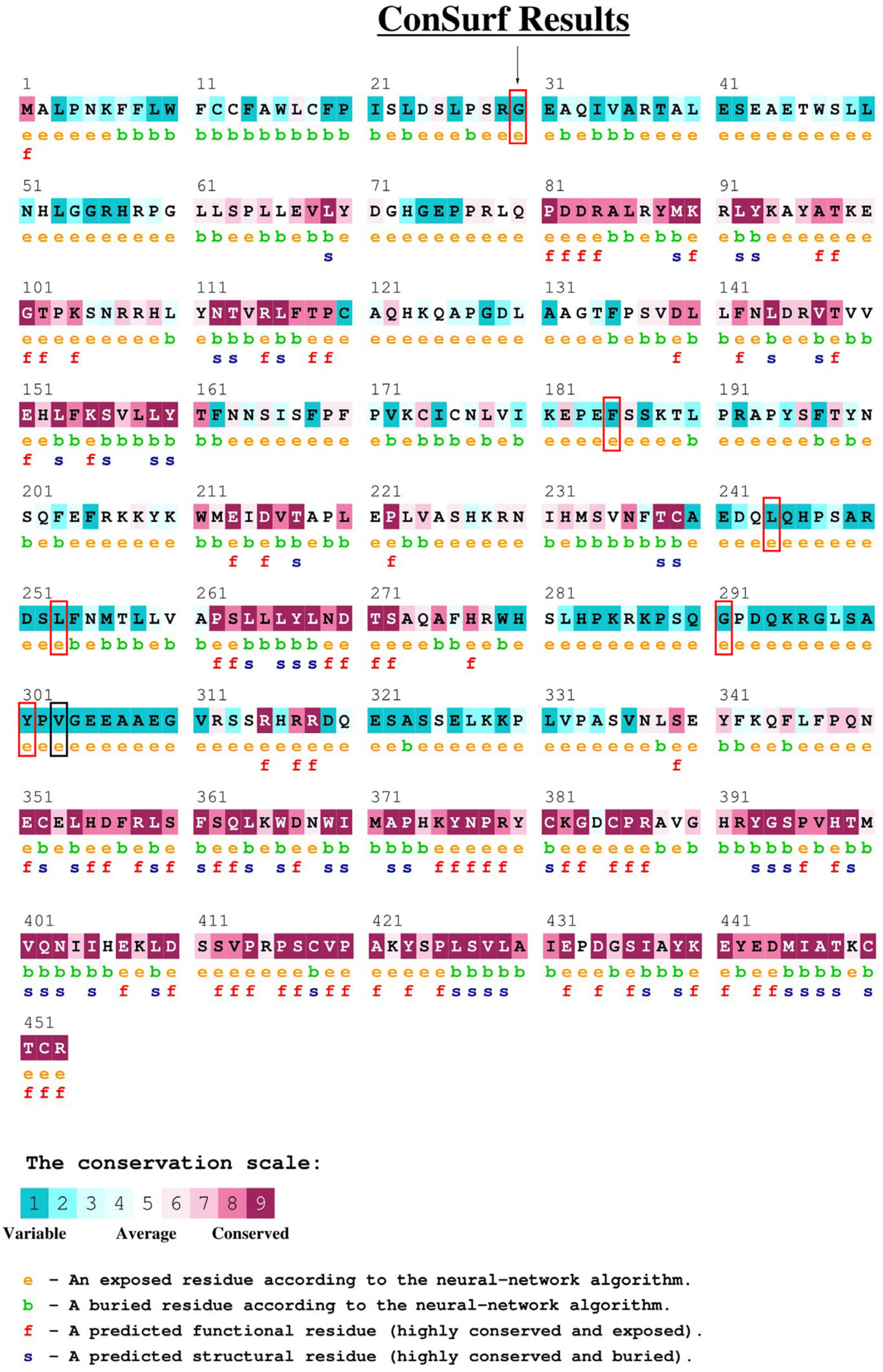

Supplementary Figure 4: ConSurf output of GDF9, using the UniRef90 protein database. Colors of the ConSurf output indicate the level of sequence conservation. Purple indicates conservation and blue indicates variability. Residues are predicted to be exposed (**e**), buried (**b**), highly conserved and exposed (**f**), or highly conserved and buried, (**-**).

Supplementary Table 1: NCBI GenBank accession numbers for BMP2, BMP4, BMP15 and GDF9 data sets. List of species and NCBI GenBank accession numbers for sequences used to construct the datasets for hypothesis testing. Species and accession numbers for each dataset are grouped together on the table. See_Supplementary_Table 1
