# Supplementary material for: Positive selection drives the evolution of endocrine regulatory bone morphogenetic protein system in mammals
Source: Oncotarget. 2018 Jan 13;9(26):18435–45. doi: 10.18632/oncotarget.24240 (PMC5915083; doi:10.18632/oncotarget.24240)
Supplement: Supplementary file 2 [file oncotarget-09-18435-s002.docx]

Table S1: NCBI GenBank accession numbers for BMP2, BMP4, BMP15 and GDF9 data sets. List of species and NCBI GenBank accession numbers for sequences used to construct the datasets for hypothesis testing. Species and accession numbers for each dataset are grouped together on the table.

|  | BMP2 |  | BMP4 |  | BMP15 |  | GDF9 |
| --- | --- | --- | --- | --- | --- | --- | --- |
| Species | Accession Number | Species | Accession Number | Species | Accession Number | Species | Accession Number |
| *Homo sapiens* | NM_001200.3 | *Homo sapiens* | NM_001202.4 | *Homo sapiens* | NM_005448.2 | *Ovis aries* | NM_001142888.2 |
| *Rattus norvegicus* | NM_017178.1 | *Rattus norvegicus* | NM_012827.2 | *Rattus norvegicus* | NM_021670.1 | *Mus musculus* | NM_008110.2 |
| *Mus musculus* | NM_007553.3 | *Mus musculus* | NM_007554.3 | *Mus musculus* | NM_009757.4 | *Capra hircus* | NM_001285708.1 |
| *Oryctolagus cuniculus* | NM_001082650.1 | *Oryctolagus cuniculus* | NM_001195723.1 | *Ovis aries* | NM_001114767.1 | *Bos taurus* | NM_174681.2 |
| *Bos taurus* | NM_001099141.1 | *Bos taurus* | NM_001045877.1 | *Bos taurus* | NM_001031752.1 | *Sus scrofa* | NM_001001909.1 |
| *Sus scrofa* | NM_001195399.1 | *Sus scrofa* | NM_001101031.2 | *Sus scrofa* | NM_001005155.1 | *Rattus norvegicus* | NM_021672.1 |
| *Nomascus leucogenys* | XM_003252488.2 | *Nannospalax galili* | XM_008839552.2 | *Bubalus bubalis* | XM_006059485.1 | *Felis catus* | NM_001165900.1 |
| *Dasypus novemcinctus* | XM_004470687.2 | *Miniopterus natalensis* | XM_016198131.1 | *pigtailed macaque* | XM_011761998.1 | *Macaca nemestrina* | XM_011716609.1 |
| *Loxodonta africana* | XM_003411553.2 | *Myotis davidii* | XM_006767704.2 | *Papio anubis* | XM_003917722.2 | *Cercocebus atys* | XM_012090234.1 |
| *Sarcophilus harrisii* | XM_012540148.1 | *Vicugna pacos* | XM_015235487.1 | *Chlorocebus sabaeus* | XM_007991731.1 | *Bubalus bubalis* | XM_006050555.1 |
| *Canis lupus* | XM_534351.5 | *Canis lupus* | NM_001287170.1 | *Panthera tigris altaica* | XM_007090093.1 | *Ailuropoda melanoleuca* | FJ546460.1 |
| *Dipodomys ordii* | XM_013010628.1 | *Acinonyx jubatus* | XM_015081200.1 | *Pan paniscus* | XM_003805950.2 | *Callithrix jacchus* | XM_002744603.3 |
| *Sorex araneus* | XM_004610935.1 | *Cavia porcellus* | XM_005004545.2 | *Sorex araneus* | XM_003640274.3 | *Papio anubis* | NM_001168763.1 |
| *Condylura cristata* | XM_004687183.2 | *Condylura cristata* | XM_004681862.1 | *Canis lupus familiaris* | XM_003640274.3 | *Nannospalax galili* | XM_008849331.1 |
| *Microcebus murinus* | XM_012755275.1 | *Sarcophilus harrisii* | XM_003758831.2 | *Echinops telfairi* | XM_004713136.2 | *Manis javanica* | XM_017652511.1 |
| *Ovis aries* | XM_004014353.3 | *Ochotona princeps* | XM_004584832.2 | *Capra hircus* | NM_001285588.1 | *Cebus capucinus* | XM_017532429.1 |
| *Capra hircus* | NM_001287564.1 | *Otolemur garnettii* | XM_012806585.1 | *Sorex araneus* | XM_004606466.2 | *Monodelphis domestica* | XM_001371982.3 |
| *Pan troglodytes* | XM_514508.3 | *Panthera tigris* | XM_007083335.2 | *Condylura cristata* | XM_004690047.2 | *Erinaceus europaeus* | XM_007519919.2 |
| *Panthera tigris* | XM_007086823.1 | *Tarsius syrichta* | XM_008050369.1 | *Felis catus* | NM_001165898.1 | *Rousettus aegyptiacus* | XM_016118390.1 |
| *Tarsius syrichta* | XM_008067106.1 | *Felis catus* | XM_006932864.2 | *Cebus capucinus imitator* | XM_017514205.1 | *Peromyscus maniculatus* | XM_006995887.2 |
| *Mesocricetus auratus* | XM_005068676.2 | *Colobus angolensis* | XM_011961818.1 | *Lipotes vexillifer* | XM_007450928.1 | *Macaca fascicularis* | XM_005557754.2 |
| *Lipotes vexillifer* | XM_007469122.1 | *Cercocebus atys* | XM_012061853.1 | *Tarsius syrichta* | XM_008055934.1 | *Vicugna pacos* | XM_006212821.2 |
| *Galeopterus variegatus* | XM_008594107.1 | *Microtus ochrogaster* | XM_005355375.1 | *Callithrix jacchus* | XM_002762884.2 | *Oryctolagus_cuniculus* | NM_001171350.1 |
| *Chlorocebus sabaeus* | XM_008018529.1 | *Equus caballus* | NM_001163970.1 | *Pongo abelii* | XM_002831651.2 | *Equus asinus* | XM_014856804.1 |
| *Callithrix jacchus* | XM_002747345.4 | *Ovis aries* | NM_001110277.1 | *Ailuropoda melanoleuca* | XM_002930506.2 | *Equus caballus* | XM_001504427.3 |
| *Pongo abelii* | XM_002829947.2 | *Papio anubis* | NM_001169087.1 | *Monodelphis domestica* | XM_007507736.2 | *Camelus ferus* | XM_006179602.1 |
| *Bison bison bison* | XM_010836460.1 | *Camelus ferus* | XM_006185422.1 | *Miniopterus natalensis* | XM_016218522.1 | *Bos mutus* | XM_005890431.2 |
| *Felis catus* | XM_003983769.3 | *Galeopterus variegatus* | XM_008570118.1 | *Pteropus alecto* | XM_006913404.1 | *Canis lupus* | NM_001168013.1 |
| *Pteropus vampyrus* | XM_011370099.1 | *Bubalus bubalis* | XM_006076290.1 | *Myotis davidii* | XM_006754222.2 | *Cavia porcellus* | XM_003464506.3 |
| *Mandrillus leucophaeus* | XM_011998522.1 | *Fukomys damarensis* | XM_010632543.1 | *Vicugna pacos* | XM_006217832.2 | *Mesocricetus auratus* | XM_013111773.1 |
| *Colobus angolensis* | XM_011941006.1 | *Tarsius syrichta* | XM_008050369.1 | *Oryctolagus cuniculus* | NM_001199117.1 | *Hmo sapiens* | NM_005260.5 |
| *Cercocebus atys* | XM_012052107.1 | |  | *Acinonyx jubatus* | XM_015074758.1 | |  |
| *Vicugna pacos* | XM_006207373.2 | |  | *Equus asinus* | XM_014827162.1 | |  |
| *Nomascus leucogenys* | XM_003252488.2 | |  | *Mustela putorius furo* | XM_004755127.2 | |  |
| *Erinaceus europaeus* | XM_007539573.1 | |  | *Propithecus coquereli* | XM_012642808.1 | |  |
| *Equus caballus* | XM_001493895.4 | |  | *Camelus dromedarius* | XM_010998065.1 | |  |
